# Anti-PD-1/Anti-PD-L1 Drugs and Radiation Therapy: Combinations and Optimization Strategies

**DOI:** 10.3390/cancers13194893

**Published:** 2021-09-29

**Authors:** Jihane Boustani, Benoît Lecoester, Jérémy Baude, Charlène Latour, Olivier Adotevi, Céline Mirjolet, Gilles Truc

**Affiliations:** 1 Department of Radiation Oncology, Centre Georges François Leclerc, UNICANCER, 21079 Dijon, France; jboustani@chu-besancon.fr (J.B.); jbaude@cgfl.fr (J.B.); clatour@cgfl.fr (C.L.); gtruc@cgfl.fr (G.T.); 2 Department of Radiation Oncology, University Hospital of Besançon, 25000 Besançon, France; 3 INSERM, EFS BFC, UMR1098, RIGHT, Interactions Greffon-Hôte-Tumeur/Ingénierie Cellulaire et Génique, University of Bourgogne Franche-Comté, 25000 Besançon, France; benoit.lecoester@edu.univ-fcomte.fr (B.L.); olivier.adotevi@univ-fcomte.fr (O.A.); 4 INSERM UMR 1231, Cadir Team, 21000 Dijon, France; 5 Department of Medical Oncology, University Hospital of Besançon, 25000 Besançon, France

**Keywords:** PD-1/PD-L1 blockade, radiation therapy, optimization strategies

## Abstract

**Simple Summary:**

Although immune checkpoint blockade has yielded unprecedented and durable responses in cancer patients, the efficacy of this treatment remains limited. Radiation therapy can induce immunogenic cell death that contributes to the local efficacy of irradiation. However, radiation-induced systemic responses are scarce. Studies combining radiation with checkpoint inhibitors suggest a synergistic potential of this strategy. In this review, we focused on parameters that can be optimized to enhance the anti-tumor immune response that results from this association, in order to achieve data on dose, fractionation, target volume, lymph nodes sparing, radiation particles, and other immunomodulatory agents. These factors should be considered in future trials for better clinical outcomes. To this end, we discussed the main preclinical and clinical data available to optimize the efficacy of the treatment combination.

**Abstract:**

Immune checkpoint inhibitors have been associated with long-term complete responses leading to improved overall survival in several cancer types. However, these novel immunotherapies are only effective in a small proportion of patients, and therapeutic resistance represents a major limitation in clinical practice. As with chemotherapy, there is substantial evidence that radiation therapy promotes anti-tumor immune responses that can enhance systemic responses to immune checkpoint inhibitors. In this review, we discuss the main preclinical and clinical evidence on strategies that can lead to an enhanced response to PD-1/PD-L1 blockade in combination with radiation therapy. We focused on central issues in optimizing radiation therapy, such as the optimal dose and fractionation for improving the therapeutic ratio, as well as the impact on immune and clinical responses of dose rate, target volume, lymph nodes irradiation, and type of radiation particle. We explored the addition of a third immunomodulatory agent to the combination such as other checkpoint inhibitors, chemotherapy, and treatment targeting the tumor microenvironment components. The strategies described in this review provide a lead for future clinical trials.

## 1. Introduction

Programmed Cell Death Protein 1 (PD-1) and its ligand Programmed Cell Death Ligand 1 (PD-L1) play a central role in inhibiting immune responses to tumor cells by reducing the activation, the proliferation, and the cytotoxic activity of T-cells [1]. The inhibition of PD-1 or PD-L1 was associated with the restoration of an effective immune response against cancer cells [2]. In patients, monoclonal antibodies blocking the PD-1/PD-L1 pathway have recently only been associated with good response rates in a minority of patients with metastatic cancers [3]. In non-responding patients, the tumor microenvironment (TME) has low levels of tumor-infiltrating CD8+ T cells, low PD-L1 expression, high cell proliferation, and low mutational burden [4]. Thus, searching for combinatorial therapies that increase responses was necessary. Radiation therapy (RT) is known to induce tumor-cell killing by creating DNA lesions. It can also induce an anti-tumor immune response [5] by enhancing the immunogenicity of tumors [6,7] and stimulating the accumulation and activation of CD8+ T cells [8]. Therefore, it was suggested that RT might increase response rates when combined to immune checkpoint therapy both in preclinical and clinical studies.

**Figure 1:**
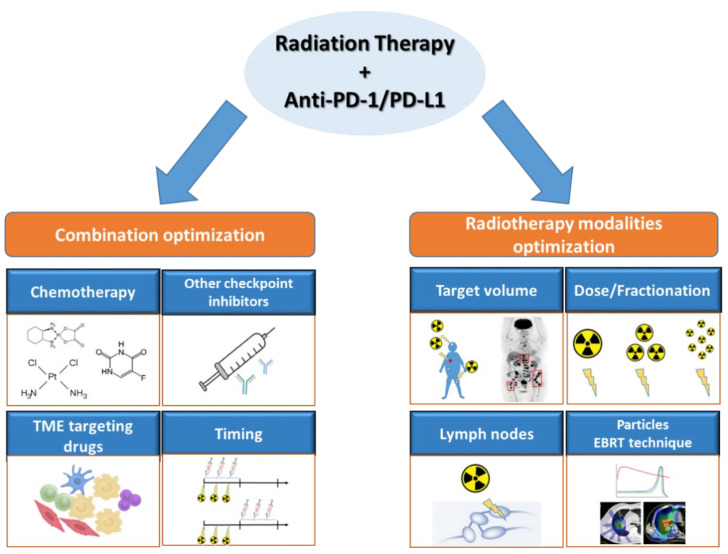
Optimization parameters for the association of radiation therapy and PD-1/PD-L1 blockade. This figure summarizes the title of the different points developed in this review. Abbreviations. TME: tumor microenvironment; EBRT: external beam radiation therapy.

Here, we reviewed multiple strategies to optimize the combination of RT and immune checkpoint inhibitors (ICI) that target the PD-1/PD-L1 axis (Figure 1). These include optimizing dose and fractionation, dose rate, target volume, radiation type (e.g., photon vs. charged particles), timing, and adding a third immunomodulatory agent. The first part of this review will describe results of preclinical studies. The second part will compile results from clinical trials evaluating different combination optimization parameters.

## 2. Radiation Therapy/Anti-PD (L)-1 Drugs Combination Optimization in the Preclinical Setting

### 2.1. Radiation Therapy Parameters Optimization

#### 2.1.1. Dose and Fractionation

In the past few years, numerous studies have investigated different ionizing radiation doses and fractionation regimens in association with anti-PD(L)-1 drugs in murine tumor models. The main purpose was to reach an optimal time-course that can induce both local and systemic anti-tumor responses.

First, studies showed the interest of combining ICI with normo-fractionated RT that corresponds to daily fractions of 1.8 to 2 Gy. In a mouse model of colon cancer (CT26), 5 × 2 Gy resulted in a T-cell infiltration in the irradiated site, and an activation of CD8^+^ T-cells producing IFN-γ, leading to an upregulation of PD-L1 within the TME [9]. The addition of anti-PD-1 or anti-PD-L1 antibodies did not only improve local control, but also had a systemic action. Mice that underwent a complete response rejected the tumor after rechallenge, highlighting a vaccine-like effect of RT and a specific memory response against the tumor [10]. By delivering fractions of 2.5 Gy or more, hypo-fractionated RT has also proved to induce responses in association with anti-PD-(L)-1. In a mouse model of colon cancer (CT26), Grapin et al. demonstrated that 18 × 2 Gy and 3 × 8 Gy regimens led to a longer tumor growth delay compared to the 1 × 16.4 Gy regimen [11]. Additionally, the local immune response was different depending on the fractionation. Indeed, while 18 × 2 Gy induced a myeloid response with an increase in myeloid-derived suppressor cells (MDSC) and tumor-associated macrophages 2 (TAM2), 3 × 8 Gy and 1 × 16.4 Gy induced a lymphoid response with an increase in CD8^+^ T-cells and regulatory T-cells (Treg) [11]. In melanoma and breast cancer models, the combination of hypo-fractionated regimen of 3 × 9.18 Gy in 3 or 5 days, and 5 × 6.43 Gy in 10 days with an anti-PD-1 antibody, resulted in growth inhibition of both irradiated primary and non-irradiated tumors [12]. Interestingly, tumor infiltrated lymphocytes (TILs) and local and systemic CD8^+^ T-cells levels were similar, regardless of the dose or the total duration of the treatment [12]. Combining an anti-PD-L1 antibody with brachytherapy delivering 3 × 8 Gy in a colorectal carcinoma model (MC38) also showed an abscopal effect, a distant effect of RT on non-irradiated lesions [13]. Furthermore, single ablative RT doses also showed promising results. Filatenkov et al. investigated the immune mechanisms contributing to complete remissions in both CT26 and MC38 colon carcinomas [14]. A single fraction of 30 Gy resulted in an intense CD8^+^ T-cells infiltration, and a loss of myeloid-derived suppressor cells (MDSC). In breast (TUBO) and colon (MC38) cancer models, 1 × 12 Gy or 1 × 20 Gy increased the level of PD-L1 expression, and the combination of those regimens with an anti-PD-L1 antibody resulted in an efficient tumor control on both irradiated and non-irradiated lesions [15]. In contrast, Vanpouille-Box et al. have demonstrated that doses per fraction greater than 12 Gy reduced the tumor immunogenicity and the abscopal effect [16]. Finally, a new RT paradigm is being evaluated, consisting of a limited number of high-dose fractions separated in time by weeks or months instead of the classic daily fractions. This new scheme, known as personalized ultra-fractionated stereotactic adaptive radiation therapy (PULSAR), was tested with an anti-PD-L1 in a MC38 colon cancer model [17]. A single dose of 16 Gy and 2 fractions of 8 Gy interspaced by 1, 4, or 10 days were compared, in addition to an anti-PD-L1 antibody. Spacing fractions by 10 days led to a better tumor control than daily fractions [17].

The current preclinical data demonstrate a wide diversity of responses of RT with anti-PD-(L)1 depending on the dose and fractionation, but also on the tumor model. Although the majority of studies evaluate the effect of hypofractionated regimens, interestingly, the use of normo-fractionated regimens still shows relevance. The optimal time-course, if it exists at all, is still to be defined.

#### 2.1.2. Target Volume and Radiation Therapy Techniques

The immunoregulatory potential of RT can be influenced, not only by the dose-fractionation, but also by the irradiated volume, and the RT technique [18]. For instance, high-dose stereotactic RT can lead to immune suppressive mechanisms such as an increase in MDSC locally and peripherally, a decrease in lymphocytes at the primary sites, and an up-regulation of Treg [19]. In some cases, the tumor may be too large to be completely irradiated, and full irradiation of these bulky tumors could be toxic. Several unconventional RT techniques have been proposed to partially treat these tumors [18]. First, the spatially fractionated RT (GRID) was designed to treat large tumors while sparing skin by delivering a heterogeneous high dose through a GRID block [18] and later through modern linear accelerators with a MultiLeaf Collimator (MLC-based GRID) [20]. Second, LATTICE radiation (LRT) can deliver high doses of RT to different areas within bulky tumors while sparing surrounding healthy organs [21]. Third, the stereotactic body radiation therapy–based partial tumor irradiation technique can target specifically the hypoxic parts of bulky tumors (SBRT-PATHY) [18]. These approaches allow high doses to be delivered into the tumor while limiting the exposure of healthy tissues and the immune system in the periphery of the tumor. Markovsky et al. reported in a preclinical trial that irradiating 50% or 100% of the tumor in an immunocompetent mouse induced the same delay in tumor growth [22]. However, this effect was abrogated in nude mice as well as in immunodeficient CD8+ mice. They also highlighted the importance of lymph nodes (LNs) in long-term tumor control, as their irradiation led to a loss of local control [22]. In another preclinical study of LRT, it was shown that partial irradiation of two vesicles of 10% of the tumor was as effective as total irradiation. This partial irradiation significantly delays tumor growth locally, probably by a bystander effect, and at a distance by an abscopal effect [23]. These preclinical results demonstrate that partial irradiation of the tumor might be sufficient to control its growth through an immunostimulatory mechanism. Thus, partial irradiation would limit the dose delivered to healthy tissues, LNs, and the immune system, which are essential for a long-term response as well as for the bystander and abscopal effects, especially when combining RT with ICI.

The irradiation of secondary lymphoid organs might have an impact on the number and function of immune cells that can be recruited to initiate the anti-tumor immune response. The role of the tumor-DLN (draining lymph nodes) in the anti-tumor T-cell activation has been evaluated in preclinical studies that demonstrated a reduced local control following tumor-DLN irradiation, genetic deficiency, or surgical removal [24,25,26]. Buchwald et al. suggested that the stem-like CD8+ T-cells in the tumor are supplied by the tumor-DLN and reduced after DLN irradiation [26]. Stem-like CD8+ T cells differentiate into terminally differentiated effectors, which have the potential for tumor-cell killing following anti-PD-L1 antibody [27]. More recently, Marciscano et al. showed that, as compared to tumor irradiation alone, irradiation of the tumor-DLN attenuated chemokine expression leading to impaired trafficking of CD8+ T cells into the TME and, ultimately, decreased survival [24]. Furthermore, elective nodal irradiation reduced the combinatorial efficacy between RT and immunotherapy.

#### 2.1.3. Dose Rate

In external beam radiation therapy, the dose rate can be changed using two different methods. The first one uses a standard LINAC with a flattening filter free (FFF) technique, in order to increase the dose rate by approximately 4 to 5 times (~2000 Monitor Unit/min vs. ~400 Monitor Unit/min). It relies on a beam with a non-uniform dose profile characterized by reduced head dispersion, foliar transmission, energy variation in a lateral direction, and reduced peripheral dose compared to a flattened beam [28]. This decreases the beam activation time, which reduces the duration of RT sessions and minimizes intra-fraction movement of the patient and/or the tumor, thus improving the reliability of the initial treatment planning [29]. The second method is FLASH therapy, which allows delivery at very high speed (several tens of grays in less than a second). This technique is in full development as it has the advantage of limiting toxicities by sparing healthy tissues [30,31]. Few studies have investigated the effect of dose rate modulation on the RT-induced immune response. Recently, using the CT26 model, we demonstrated no influence of a moderately high-dose rate using the FFF technique on the anti-tumor immune response [32]. These results need to be confirmed by clinical data. However, these preliminary results may encourage RT oncologists to increase the dose rates of their treatment, even in combination with immunotherapy. To our knowledge, there is currently no preclinical data concerning FLASH therapy and its combination with anti-PD-(L)-1. However, the scientific enthusiasm for this technique prompts the development of such studies.

#### 2.1.4. Particles

Almost all data regarding RT-induced immune response were obtained with photon-based ionizing radiation. Charged-particle therapy (CPT) with protons or heavier ions was described to induce immune responses. Most of the studies evaluated components related to the immune response on tumor cells in vitro, essentially using carbon ions, rather than the modification of the TME in vivo. CPT presents specific physical and biological properties, such as the differential induction of cell death, and dosimetric properties which allow the sparing of healthy tissues such as DLN and circulating immune cells to reduce RT-induced lymphopenia [33,34]. Preliminary animal studies have shown that CPT induced abscopal responses after irradiation of the primary tumor [34,35]. However, a direct comparison of conventional RT and CPT is required. Recently, Helm et al. found that X-rays and carbon ion have the same efficacy when combined with anti-PD-L1 and anti-CTLA-4 antibodies, both on the irradiated tumor and on non-irradiated lesions [36]. Based on in vitro experiments, Permata et al. have shown that the expression of PD-L1 mRNA and protein increased more importantly after high linear energy transfer (LET) carbon ion irradiation than after photon based-RT [37]. This induction might be associated with a better sensitivity to anti-PD-L1 antibody, which needs to be evaluated in vivo. In parallel, Spina et al. evaluated the effects of carbon-ion irradiation on immune modulation using orthotopic 4T1 mammary tumors [38]. They found a higher induction of pro-inflammatory cytokines after carbon ions than after photons [38]. In another study, carbon ion radiation induced an immune cell death characterized by the secretion of high mobility group box 1 (HMGB1) in human cancer cell lines [39]. The levels of HMGB1 were similar with equivalent doses of photon irradiation [39]. Finally, our team has recently described the radiation-induced immune response with a single fraction of proton on a colorectal tumor model. We described a significant T cell (CD8+ and CD4+), TAM1 and Treg infiltration [40]. Using a transcriptomic analysis, we have also highlighted the activation of type 1 interferon pathway.

In conclusion, these studies suggest that charged particles may be more immunogenic than photons. Thus, their combination with ICI is of great interest.

### 2.2. Radiation Therapy/Anti-PD-(L)-1 Combination Timing

There are many pieces of evidence that ionizing radiation induces an upregulation of PD-L1 in the tumor [9,15,41], suggesting that an adjuvant blockade of the PD-1/PD-L1 axis after RT is relevant. In a breast murine model (TUBO), Liang et al. reported that a single injection of anti-PD-L1 antibody 21 days after 1 × 15 Gy resulted in a tumor regression with tumor rejection in most mice, contrary to RT alone [42]. Conversely, the sequential association of RT and anti-PD-L1 antibody (5 × 2 Gy followed by an injection of anti-PD-L1 at day 7) failed to improve survival in mice with colon cancer (CT26) over RT alone, whereas the injection of anti-PD-L1 antibody at day 1 or day 5 after the beginning of RT did [9]. In a pancreatic murine model (KPC), simultaneous (days 0, 3, 6, and 9) addition of anti-PD-L1 antibody to one fraction of 12 Gy was found to be more efficient on tumor growth than the sequential schedule (anti-PD-L1 at days 6, 9, 12, and 15) [43]. A combination of concurrent anti-PD-L1 antibody (days 0, 2, and 4) with a fraction of 10 Gy also improved survival of mice with glioma (GL261) compared to either therapy alone [44]. Similar evidence supporting a synergetic effect of the concurrent combination of anti-PD-L1 antibody and a fraction of 10 Gy were found in a murine head and neck cancer model (B4B8) [41]. More recently, Moore et al. compared three different timings between RT and anti-PD-L1 antibody in a colon cancer model (MC38) [17]. Even if there was a benefit of the addition of immunotherapy when given after the single fraction of 16 Gy (days 2, 3, 4, and 6), the optimum combination was found to be the concurrent one (four injections of ICI with RT at day 2). However, when anti-PD-L1 antibody was given before RT (four daily injections followed by the single fraction of 16 Gy at day 4), no additive effect was found compared to RT alone. In parallel, another study showed consistent results with the use of neoadjuvant anti-PD-1 antibody [45]. Interestingly, the administration of anti-PD-1 antibody prior to RT resulted in an increased radiosensitivity and death of CD8+ T cells, which could explain the suboptimal effect of neoadjuvant ICI followed by RT, compared to concomitant or adjuvant ICI [45].

Therefore, studies evaluating the right timing to combine RT and anti-PD-(L)-1 lead to conflicting results. One thing is for sure, timing has an impact on efficacy, and further data are warranted.

### 2.3. Combination with Other Therapies

#### 2.3.1. Targeting Molecular Modulators of the Immune Response

In a preclinical model of melanoma, Twyman-Saint Victor et al. found that the resistance to RT plus anti-CTLA-4 was due to T-cell exhaustion, notably mediated by PD-1/PD-L1 axis activation with an upregulation of PD-L1 on melanoma cells. Hence, there was a significant improvement of survival in the dual checkpoint blockade (anti PD-L1 plus anti-CTLA-4 antibodies) group associated with RT (1 × 20 Gy) as compared to RT plus either antibody [46]. Addition of PD-L1 blockade reversed T-cell exhaustion, increased proliferation of TIL, and enhanced oligoclonal T-cell expansion [46,47]. Oweida et al. showed that the immune checkpoint receptor T-cell immunoglobulin and mucin-domain containing-3 (TIM-3), was upregulated on TIL CD8+ T cells and Treg after radiation (1 × 10 Gy) plus PD-L1 blockade in head and neck squamous cell carcinoma orthotopic tumor bearing mice (LY2 and MOC2) [48]. Adding anti-TIM-3 significantly reduced tumor growth, enhanced T-cell cytotoxicity, and decreased Treg infiltration. Despite these anti-tumor effects induced by the triple combination, there was no long-term tumor rejection [48]. Moreover, RT (3 × 8 Gy) plus anti-PD-1 antibody combined with an antibody targeting T-cell immunoreceptor with Ig and ITIM domains (TIGIT) led to a significant anti-tumor effect in CT26 and B16-F10 models, which was less effective with RT and either antibody alone [11]. Interestingly, the intake of this anti-TIGIT was lost when RT was administered with a normo-fractionated regimen. Optimization of fractionation would therefore play a central role in increasing the efficiency of these combinations.

4-1BB (CD137), an activation-induced costimulatory molecule, is an important regulator of immune responses through the activation of cytotoxic T lymphocytes and the production of high amounts of IFN-γ. Stimulation of 4-1BB with agonist antibodies is a promising strategy to improve the therapeutic efficacy of ICI or to overcome the resistance to ICI. Rodríguez-Ruiz et al. demonstrated in melanoma, breast, and colon cancer mouse models, that mice receiving RT (3 × 8 Gy) with anti-PD-1 and anti-4-1BB antibodies achieved faster and almost constant complete responses on distant non-irradiated tumor lesions [49]. Importantly, all cured mice were fully protected against subsequent tumor rechallenge. This abscopal effect was dependent on type I IFNs pathway, BAFT3 dendritic cells, and CD8+ T cells [49]. In addition, another preclinical study has shown that TGFβ played a role in the modulation of the response to RT, and that TGFβ blockade with RT enhanced dendritic cells activation and induced CD8+ T-cell responses to endogenous tumor antigens [50]. TGFβ blockade added to radio-immunotherapy (local RT + anti 41BB + anti-PD-1) notably strengthened the abscopal effect by inducing TIL in distant non-irradiated tumors [51]. More recently, Lan et al. reported the development of bintrafusp alfa, a bifunctional fusion protein composed of the extracellular domain of the TGFβ RII receptor to trap TGFβ, fused to a human immunoglobulin G1 antibody blocking PD-L1 [52]. Using this molecule in combination with RT in several immune-cold murine tumor models, they showed a synergic action leading to higher survival, increased TIL, reprogramming of the TME, and reduced tissue fibrosis [53]. Additionally, regression of spontaneous lung metastases was observed. This abscopal effect was attributed to an enhanced influx and activation of cytotoxic CD8+ T cells and reduction in immunosuppressive cells in premetastatic lung niches.

Among new targets, the glucocorticoid-induced TNFR-related protein (GITR) is known to potentiate the response to tumors by co-activating T-cells and NK cells effectors, modulating dendritic cells functions and inhibiting Treg [54]. In an anti-PD-1 antibody resistant tumor 344SQR murine model, combination of anti-GITR antibody, anti-PD-1 antibody, and RT (3 × 12 Gy) significantly improved survival and abscopal response, with 50% of tumor-free mice [55]. Although targeting immune checkpoints in their extracellular part is widely used, targeting the transduction pathways induced by their activation is another possibility. SH2-containing protein tyrosine phosphatase-2 (SHP2) is an oncogenic phosphatase known to facilitate growth and survival signaling downstream of numerous receptor inputs. Addition of the SHP-2 inhibitor SHP099 to radio-immunotherapy (RT 3 × 12 Gy + anti-PD-1 antibody) improved the anti-tumor response in an anti-PD-1 antibody-resistant lung cancer model [56].

Many molecules targeting immunosuppressive mechanisms are in development. Assessing their effects in combination with RT and anti-PD-(L)-1 seems paramount.

#### 2.3.2. Targeting Immune Suppressive Cells

One major therapeutic obstacle when dealing with immunotherapy is the immunosuppressive TME that can result from an increased immunosuppressive cells infiltration such as Treg or TAM2. In a mouse model of human papillomavirus (HPV)-associated head and neck cancer, Newton et al. combined anti-PD-1 and anti-CTLA-4 antibodies with RT (2 × 10 Gy). Two drugs that selectively deplete Treg [57], cyclophosphamide and a small-molecule inducible nitric oxide synthase (iNOS) inhibitor, L-NIL [58], were added. Only the combination of cyclophosphamide and L-NIL with dual checkpoint inhibition and RT induced the rejection of 70% of tumors. The anti-tumor activity was CD8^+^ T cell-dependent and led to an immunologic memory against tumor-associated HPV antigens [57]. On the other hand, indoleamine-pyrrole 2,3-dioxygenase (IDO1) catabolized tryptophan required for lymphocyte maintenance and function, into kynurenine. Thus, IDO1 suppresses T cell activation and promotes Treg expansion [59,60]. Ladomersky et al. developed a triple therapy based on the association of anti-PD-1 antibody, a whole brain RT (5 × 2 Gy) and an oral gavage with IDO1 inhibitor BGB-5777 in a glioma model [61]. Survival was significantly improved compared to RT alone or with either therapy [61]. Furthermore, RT stimulates CSF-1 secretion by tumor cells allowing TAM2 recruitment. TAM2 produces factors and cytokines that promote the development of Treg, metastasis, angiogenesis, matrix remodeling, and PD-1 expression [62,63]. Therefore, Jones et al. tested a combination of intraperitoneal-injected therapy targeting CSF-1 with anti-PD-L1 antibody and RT (1 × 10 Gy) [64]. In pancreatic tumors resistant to immune checkpoint blockade, the triple combination therapy led to a greater tumor regression than with monotherapy or combined therapies. These results were not observed in the MC38 model that was sensitive to the combination of anti-PD-L1 antibody and RT without anti-CSF1, suggesting preferential use in resistant tumors [64]. MER proto-oncogene tyrosine kinase (MerTK), a macrophage-specific phagocytic receptor, also represents a promising target, as its overexpression has been linked with poor prognosis. Anti-MerTK antibodies have shown their interest, by promoting pro-inflammatory effects of immunogenic cell death, resulting in the suppression of the phagocytosis of apoptotic bodies after RT [65]. Inhibition of the MerTK in combination with anti-PD-1 antibody and stereotactic RT (3x12 Gy) significantly delayed tumor growth of non-irradiated lesions and reduced numbers of lung metastases in mice with bilateral lung adenocarcinoma xenografts. Furthermore, triple combination promoted the upregulation of CD8+ CD103+ tissue-resident memory cells, known to correlate with good prognosis [66,67].

#### 2.3.3. Priming, Recruitment, and Activation of Dendritic cells

Intra-tumoral BATF3-expressing dendritic cells correlates with a T cell-inflamed TME and ICI efficacy, suggesting the importance of T-cell priming and cross presentation in the response to immunotherapy [18,19,68,69]. To increase the priming of T cells in the DLN, Hammerich et al. used an in situ injection of FLT3L and polyICLC with RT (1 × 10 Gy) and anti-PD-1 antibody in lymphoma tumor mice model (A20 cells) [70]. FLT3L triggers the recruitment of FLT3+ dendritic cells, while PolyICLC allows the activation of TLR3+ dendritic cells. This resulted in delayed tumor growth compared with in situ injection alone, and durable remissions increased from 40% to 80% in mice [70].

#### 2.3.4. Combination with Chemotherapy

Kroon et al. found that cisplatin considerably potentiated the abscopal effect of radio-immunotherapy consisting of a single fraction of 10 Gy, four injections of anti-PD-1 antibody, and anti-4-1BB antibody in AT3 mouse model [71]. Furthermore, this combination increased overall survival (OS) in a CD8+ T-cell-dependent manner [71]. Another study showed that cisplatin-based chemoradiation (2 × 12Gy) combined with anti-PD-1 antibody significantly improved RT-induced abscopal effects in MC38 colon cancer and B16 melanoma models [72]. The abscopal effect in these models was based on the recruitment of CXCR3 + lymphocytes into the non-irradiated tumor secreting CXCL10 chemokines possibly through the systemic action of cisplatin [72]. Recently, Joseph et al. demonstrated in three tumor models that concurrent combination of a 5 fluorouracil-cisplatin-based chemoradiation with a dual CTLA-4 and PD-1 blockade was required to achieve an optimal anti-tumor effect and to establish a broad and long-lasting protective anti-tumor T cell immunity through the activation of CD103+ dendritic cells and intratumoral T-cell Th1 and TRM polarization [73].

#### 2.3.5. Targeting Angiogenesis

RT can induce the secretion of VEGF, an angiogenic factor expressed in cancer. Yang et al. demonstrated that STING pathway regulated tumor angiogenesis in murine Lewis lung carcinoma model [74]. The combination of STING agonist with VEGFR2 blockade induced complete tumor regression and vascular normalization. Interestingly, in tumors resistant to ICI (anti-PD-1 or anti-CLTA-4 antibodies), the addition of STING agonist and VEGFR2 blockade improved the complete response rates compared with ICI alone (anti-PD-1 or anti-CLTA-4 antibodies). These results encourage the evaluation of this triple combination with RT, as the STING pathway can be activated by irradiation [16]. Recently, VEGF blockade efficacy was confirmed in combination with radio-immunotherapy (anti-PD-L1 antibody + 4 × 10Gy) in murine Lewis lung carcinoma model [75].

Taken together, these data demonstrate that an effective stimulation of the anti-tumor response can be achieved by optimizing several parameters. These promising preclinical results served as the basis for testing the combination of RT and anti-PD(L)1 blockade in the clinical setting.

## 3. Radiation Therapy/Anti-PD(L)-1 Drugs Combination Optimization in Clinical Trials

### 3.1. Radiation Therapy Parameters Optimization

The goal of RT is to increase tumor control while sparing healthy tissue. Is it currently used to treat approximately 50% of cancer patients; immunotherapy is an integral part of the management of many advanced or metastatic tumors, but also in palliative treatments, hoping for a significant improvement in patient survival. The beneficial effects of RT on non-irradiated tumor sites known as the abscopal effect (from the Latin “ab scopus”, i.e., far from the target) have now been demonstrated. The distant activation of the immune system by irradiation appears to be markedly increased when combined with ICI [76]. If immunotherapy is seen to be more effective, it is possible that it itself has a radiosensitizing effect. These findings are at the origin of a strong enthusiasm for the association and in particular for oligometastatic or even multi metastatic patients. We already have studies for several types of tumors, in particular melanoma and non-small cell lung cancers, which show better progression-free survival rates in patients treated with ICI and RT, with acceptable toxicity. However, the data are not yet sufficiently solid, and it remains to be proven which combinations and which modifications of the RT parameters (dose, volume, particles, fractionation, etc.) can bring a therapeutic improvement. It is also necessary to ensure that these associations, whatever their modalities, are safe.

#### 3.1.1. Fractionation

Identifying the ideal RT fractionation scheme to associate with immunotherapy represents a major challenge. Several studies have found that RT can induce an anti-tumor immune response in a dose-dependent manner [77,78]. In a large National Cancer Database (NCDB) study, 5281 metastatic melanoma patients who received palliative RT were included [79]. Patients received either conventional RT (< 5 Gy/fraction) or hypofractionated RT (≥ 5 Gy/fraction) with or without immunotherapy (ICI, interleukins, or oncolytic virus). The highest OS rates were obtained in patients who received hypofractionated RT and immunotherapy [79]. In the phase 2 PEMBRO-RT study, patients with advanced non-small cell lung cancer received either pembrolizumab alone or SBRT (3 × 8 Gy) to one lesion followed by pembrolizumab within 7 days [80]. The objective response rate and survival outcomes were significantly higher with SBRT [80]. Finding the optimal dose and fractionation requires accounting for tumor pathological type, tumor size, tumor location, presence of metastases, intrinsic radiosensitivity, and host characteristics, which makes it difficult to determine the standard regimen.

#### 3.1.2. Dose

Another factor that can influence the outcomes of RT and ICI combination is the exposure to low-dose RT [81]. Preclinical studies have suggested that low-dose RT may stimulate immune cells and modulate the TME. In a post hoc analysis of ipilimumab with high-dose RT, tumors exposed to low-dose scatter RT were more likely to decrease than unexposed distant tumors [82]. A post hoc analysis [83] of three trials exploring RT and immunotherapy associations was performed, including 26 patients. There was significantly more objective responses in low-dose lesions (1–20 Gy) compared to non-irradiated lesions (< 1 Gy). Based on these observations, Arnold et al. developed a model where high-dose RT increases antigen release and presentation, and primes immune cells, whereas low-dose RT promotes immune-cell infiltration into the stroma and tumor bed [84].

Thus, a single optimal RT scheme might not exist, but rather several ones, depending on the tumor and host characteristics.

#### 3.1.3. Target Volume

Most of the clinical trials testing RT with immunotherapy have irradiated a limited number of sites, with a low impact on the systemic response [85,86]. Some authors have suggested that, in order to enhance the synergy between RT and ICI, all lesions should be irradiated [87]. This would most likely promote antigen presentation, improve immune access to tumor bed, and reduce the immunosuppressive barrier effects of bulky lesions in all areas of the disease. In a subgroup analysis of a phase III trial testing the efficacy of RT with anti-CTLA-4, it was suggested that patients who might benefit the most from RT were those with an oligometastatic disease [88]. Several studies have shown that local treatment of all sites in oligometastatic patients can significantly prolong survival without severe side effects. In Bauml et al., patients with oligometastatic (≤ 4 lesions) non-small cell lung cancer were treated with anti-PD-1 antibody and local treatment (surgery, RT, or radiofrequency ablation) for all lesions [89]. Median PFS was 18.7 months and median OS was 41.6 months. A phase I trial [90] and the phase II multicenter SABR-COMET [91,92] trial demonstrated that multisite SBRT followed by PD-1/PD-L1 antibody inhibitors resulted in high local and distant control with improved OS. Results from ongoing clinical trials allowing irradiation of all lesions are awaited (NCT03275597, NCT02523313, and NCT03391869).

On the other hand, multisite RT increases the irradiated volume and thus the risk of toxicities that can dampen the outcomes. For instance, in patients treated with radio-immunotherapy, RT-induced lymphopenia was associated with a lower response to anti-PD-1/PD-L1 drugs and a lower survival [93,94]. The depth and duration of lymphopenia depend on the location of the irradiation, the treated volumes, and the irradiation technique [95]. For instance, rotational intensity-modulated RT can deliver low doses to large volumes of healthy organs. The irradiation of large volumes can affect circulating or tissue-resident lymphocytes [96]. This should prompt clinicians to rethink the modalities of RT, especially in combination with immunotherapy. Furthermore, clinical data on partial tumor irradiation in case of high metastatic burden or bulky tumors (>65 mL) combined with pembrolizumab, could lead to similar local control compared to total irradiation [88]. It is clear that the irradiation fields play an important role and thus, sparing non-metastatic lymph nodes and reducing irradiated volumes can potentially reduce lymphopenia and improve the anti-tumor immune response [96]. Thus, the extent of irradiation and the incorporation of elective nodal irradiation could have a significant impact on outcomes. Future clinical trials investigating concurrent RT with ICI need to compare DLN irradiation vs. sparing LN, and take into account the irradiation technique and volumes.

#### 3.1.4. Particles

Conventional RT uses photons generated by electron accelerators, but high-energy charged particles such as protons and carbon-ions, can improve precision of dose delivery with better normal tissue sparing. Protons and carbon ions are considered superior to photons for distribution ballistics. Data regarding their immunologic effects remain scarce, with mostly results from in vitro assays or precliclinal models. By sparing more healthy tissue, protons appear to be an attractive therapy to combine with immunotherapy. Whether they can be more effective than conventional RT in combination with immunotherapy is unknown. Seven phase I or II trials with small samples were designed to evaluate the safety and efficacy of combining proton therapy and immunotherapy in cancer patients (NCT03765190, NCT03539198, NCT03818776, NCT04834349, NCT02444741, NCT04671667, and NCT03267836), and are currently ongoing or not yet recruiting. These trials are investigating proton therapy with anti-PD-1 or anti-PD-L1 antibodies for metastatic cancers, non-small cell lung cancer, head and neck cancer, or meningioma. On another note, a phase 2 study is evaluating the combination of anti-PD-1 antibody (pembrolizumab) with re-irradiation and intra-tumoral administration of nanoparticles designed to destroy tumor cells when activated by RT for the treatment of inoperable locoregional recurrent head and neck squamous cell cancer (NCT04834349). Charged particle therapy and immunotherapy currently represent an emerging partnership for immune activation and results from ongoing studies are eagerly awaited.

#### 3.1.5. Radiation Therapy/Anti-PD(L)-1 Combination Timing

When combining RT and ICI, timing might play a crucial role [97]. Data from the phase 3 PACIFIC trial for patients with stage III locally advanced and unresectable non-small cell lung cancer that had not progressed after ≥2 cycles of chemoradiation revealed that the addition of consolidation durvalumab (anti-PD-L1 monoclonal antibody) significantly improved PFS and OS over placebo [98,99]. Although this study did not aim to assess the timing between RT and immunotherapy, durvalumab had to start 1 to 42 days following chemoradiation. Pre-specified subgroups analysis showed an improved survival rate in patients who received the anti-PD-L1 antibody in the 14 days following chemoradiation [99]. This potentially indicates that concomitant combination therapy may be more optimal than delaying immune checkpoint blockade. In patients with advanced non-small cell lung cancer, Shaverdian et al. found that RT before pembrolizumab translated into significantly longer survival outcomes compared to pembrolizumab alone [100]. A meta-analysis evaluated stereotactic radiosurgery (SRS) for brain metastases and ICI with a focus on the optimal timing sequence [101]. Concurrent therapy was defined as the administration of ICI and SRS within one month. The OS, local control, and regional brain control rates were significantly higher with concurrent therapy. A systematic review recently reported the effectiveness and safety of concurrent treatment, defined as stereotactic radiotherapy performed within 30 days of ICI administration. This strategy led to interesting local control, especially for brain lesions, without increasing toxicity [102]. The results of the PRACTICE study suggest a possible negative impact of receiving palliative RT within 6 months prior to immunotherapy initiation in terms of disease control rate and time to treatment failure. On the contrary, the concurrent palliative RT group exhibited improved time to treatment failure periods compared with the no RT and previous palliative RT group [103].

Overall, these studies confirm the importance of the concomitant timing of RT and ICI that was suggested in preclinical studies.

Currently, more than 30 phase III clinical trials combining RT and immunotherapy are ongoing. Among these, about two thirds evaluate a concomitant +/− adjuvant combination, and a third uses the immunotherapy in induction +/− adjuvant condition [104].

### 3.2. Combination with Other Therapies

Although the combination of RT and ICI has shown synergy, clinical responses vary widely. Recent studies have implied that the addition of an immunologically active agent to RT and ICI might increase effectiveness and response rates [105]. Several clinical trials are being performed or completed to explore the combination of RT and ICI with a third immunomodulatory agent, such as IDO1 inhibitor in glioblastoma (NCT04047706), Toll-like Receptor (TLR) 7 Agonist in breast cancer with skin metastases (NCT01421017), or TLR9 Agonist in chemotherapy-refractory metastatic pancreatic adenocarcinoma (NCT04050085).

Floudas et al. assessed the association of AMP-224, an anti-PD-1 fusion protein, with low-dose cyclophosphamide, and SBRT in chemo-refractory patients with metastatic colorectal cancer [106]. The association was well tolerated, but did not increase response rates or survival. Several clinical trials are ongoing to explore the safety and efficacy of combining RT with anti-PD-1/PD-L1 drugs and chemotherapy. The phase 3 randomized international study, KUNLUN, will enroll patients with locally advanced, unresectable esophageal squamous cell carcinoma treated with definitive chemoradiation with or without concurrent Durvalumab (NCT04550260). INTERACTION is a phase 2 trial that will enroll patients with stage III squamous cell anal carcinoma to evaluate the efficacy and safety of DCF (Docetaxel, Cisplatin, and 5-fluorouracil) plus ezabenlimab, an anti-PD-1 antibody, as neoadjuvant treatment, before concurrent chemoradiation in responders (NCT04719988). Finally, in patients with metastatic anal carcinoma, the phase 2 trial SPARTANA will investigate the efficacy of combining hypofractionated RT to metastatic lesions, anti-PD-1 antibody, and DCF in terms of PFS and objective response (NCT04894370).

## 4. Conclusions

The interest in combinations based on RT and anti-PD-(L)1 drugs is now well accepted by the scientific and medical community. The doubts about the increased risk of toxicity seem to be diminishing, although attention must remain in mind.

The impact of new RT modalities on the immune response and on the efficacy of immunotherapy are currently being evaluated in preclinical conditions (FLASH, new combinations, etc.) and these results will be valuable for developing future clinical trials.

In order not to miss out on the effectiveness of these combinations, it is necessary to optimize several parameters and adapt them to each indication. We encourage investigators of future clinical trials to be aware of the importance of optimizing RT. This type of combination is certainly the key to giving RT a place as a curative treatment for metastatic patients.

The development of specific biomarkers will also be necessary for these optimization of association schemes. Their objectives will be to describe and predict the radiation-induced immune response, and thus to be able to adapt the immunotherapies which will have the best probability of being effective.

Among these biomarkers, two are under development in preclinical and clinical studies via ancillary studies to clinical trials. The first category of biomarkers is developed from tumor samples (irradiated or out-of-field) taken before and/or after treatment. Even if these biopsies can be invasive, they make it possible to analyze the tumor microenvironment with precision (by transcriptomics analysis, Single Cell, etc.) and to precisely map the radiation-induced immune response and the evolution of the expression of the target of specific immunotherapies. The second category of biomarkers is developed from liquid biopsies. They have the advantage of being easily accessible, and can analyze at several kinetic points. The aim of these biomarkers is to map the systemic response to RT, sometimes with a risk to lack of detection sensitivity. Among these are evaluated the concentrations of circulating cytokines, immune cells, activity, and specificity of LT CD8+, exosomes, etc. It is important that each clinical trial evaluating combinations of RT and immunotherapy plans to collect these biological samples in order to have a sufficient amount of data to perform bioinformatics analysis comprising a large number of variables.
